# Pilot study of a culturally sensitive intervention to promote genetic counseling for breast cancer risk

**DOI:** 10.1186/s12913-022-08193-x

**Published:** 2022-06-25

**Authors:** Vida Henderson, Jessica M. Madrigal, Le’ Chaun Kendall, Pooja Parekh, Jennifer Newsome, Ifeanyi Beverly Chukwudozie, De Lawnia Comer-Hagans, Vickii Coffey, Giesela Grumbach, Shirley Spencer, Carolyn Rodgers, Ravneet Kaur, Lara Balay, Tara Maga, Zo Ramamonjiarivelo, Catherine Balthazar, Robert Winn, Karriem Watson, Angela Odoms-Young, Kent F. Hoskins

**Affiliations:** 1 Fred Hutchinson Cancer Center, 1100 Fairview Ave. N, Seattle, WA 98109 USA; 2 University of Illinois Cancer Center, 818 S. Wolcott Ave MC 709 SRH, Chicago, IL 60612 USA; 3 University of Illinois College of Medicine, 1801 W Taylor St, Chicago, IL 60612 USA; 4 Foundation for the National Institutes of Health, 11400 Rockville Pike #600, North Bethesda, MD 20852 USA; 5 University of Texas at Austin, 1501 Red River Street, Austin, TX 78712 USA; 6 Governors State University College of Health and Human Services, 1 University Parkway, University Park, IL 60484 USA; 7 University of Illinois at Chicago College of Applied Health Sciences, 1919 W Taylor St MC517, Chicago, IL 60612 USA; 8 University of Illinois Hospital and Health Sciences System, 1801 W Taylor St, Chicago, IL 60612 USA; 9 Texas State University, 601 University Drive, San Marcos, TX 78666 USA; 10 Virginia Commonwealth University Massey Cancer Center, 401 College St Box 980037, Richmond, Virginia 23298 USA; 11 National Institutes of Health, All of Us Research Program, 200 Independence Ave, SW, Washington, DC, 20201 USA; 12 Cornell University College of Human Ecology, Martha Van Rensselaer Hall, Ithaca, NY 14853 USA; 13 University of Illinois College of Medicine, University of Illinois Hospital and Health Sciences System, 1801 W Taylor St, Chicago, IL 60612 USA

**Keywords:** Genetic counseling, Black/African American women, Disparities, Breast cancer, Decision making, Narrative intervention

## Abstract

**Background:**

Despite the benefits of genetic counseling and testing, uptake of cancer genetic services is generally low and Black/African American (Black) women are substantially less likely to receive genetic services than non-Hispanic White women. Our team developed a culturally sensitive, narrative decision aid video to promote uptake of genetic counseling among Black women at risk for a hereditary breast cancer syndrome that can be incorporated in conjunction with population-based cancer risk assessment in a clinical setting. We report here a pilot study to demonstrate changes in intention to access genetic counseling and intervention satisfaction.

**Methods:**

Black women who were personally unaffected by breast cancer and were recommended for genetic counseling based on family history screening in a mammography center were recruited at the time of the mammogram. A prospective, pre-post survey study design, guided by theoretical constructs, was used to evaluate baseline and immediate post-intervention psychosocial factors, including intention to participate in genetic counseling and intervention satisfaction.

**Results:**

Pilot recruitment goals were met (*n* = 30). Pre-intervention, 50% of participants indicated that they were extremely likely to make a genetic counseling appointment, compared with 70% post-intervention (*p* = 0.05). After watching the intervention, 50% of participants indicated that the video changed their mind regarding genetic counseling.

**Conclusions:**

This study demonstrated cultural satisfaction with a decision aid intervention designed to motivate Black women with hereditary breast cancer risk to attend a genetic counseling appointment. Our study showed that intention may be a specific and key construct to target in interventions designed to support decision-making about genetic services. Study results informed the design of a subsequent large scale, randomized implementation study.

**Trial registration:**

Trial registration: Clinicaltrials.govNCT04082117.

Registered September 9, 2019.

Retrospectively registered.

**Supplementary Information:**

The online version contains supplementary material available at 10.1186/s12913-022-08193-x.

## Introduction

Carriers of germline mutations in cancer susceptibility genes have up to an 85% lifetime risk of developing breast cancer compared with 12% for the general population [1–3]. Cancer genetic risk assessment (CGRA) and genetic counseling allow those with hereditary breast cancer risk to make informed choices about genetic testing and cancer prevention options [4]. Genetic counselors determine whether genetic testing should be performed based on pedigree analysis, provide education and pretest counseling on genetic testing risks and benefits, interpret results of genetic testing in the context of cancer risk counseling, and facilitate cascade testing of at-risk family members [5]. Although genetic counseling and testing are a critical component of personalized cancer risk management, over 90% (~ 10.7 million) of those with hereditary susceptibility to breast and ovarian cancer in the U.S. have yet to be identified and tested [1, 6]. Underutilization is especially problematic for African American women, who are 40% more likely to die from breast cancer than non-Hispanic white (NHW) women. Breast cancers arising in Black women frequently share features that are common among *BRCA1*-associated cancers, such as younger age of onset, high tumor grade, and negative hormone receptor status, which all contribute to disparate breast cancer mortality rates [3, 7–10]. The U.S. Preventive Services Task Force (USPSTF) guidelines recommend incorporating assessment of family history into routine primary healthcare in order to identify women at risk for hereditary breast and ovarian cancer so that genetic counseling and testing can be provided for women at risk for hereditary breast cancer syndromes [11, 12].

Despite the benefits of genetic counseling and testing, uptake of cancer genetic services is generally low. This is particularly problematic among Black women, who are approximately 50% less likely to receive genetic services than NHW women due to multi-level barriers, including bias in provider referrals, lack of perceived benefit, barriers to access and lack of awareness of genetic services, and cost [1, 6, 13–26]. Increasing utilization of genetic services for Black women may reduce breast cancer mortality disparities by improving early detection and disease prevention among those at greatest risk. Moreover, it will help curtail the problem of underrepresentation of racially and socioeconomically diverse populations in genetic and genomic research [1, 13, 17, 27]. Our prior work demonstrated that it is feasible to implement a population-based approach to identify candidates for genetic counseling for hereditary breast cancer risk in community health centers located in underserved minority communities. This strategy addresses systemic barriers and improves the rate of provider referrals and access to genetic counseling [28]. However, only 16% of referred women attended a genetic counseling appointment even after receiving a recommendation from their physician, prompting the need for interventions that support informed decision-making about genetic counseling services for underserved minority women.

Decision aids promote positive health behavior change, improve health outcomes and mitigate health literacy barriers. Relatively little work has been conducted to understand the decision-making process among racial/ethnic minority women recommended for genetic counseling for breast cancer risk, or to develop culturally-tailored decision aids to promote uptake of genetic services [27, 29–33]. The tools developed to date are primarily to supplement information provided in genetic counseling sessions, are largely print or telephone-based, are often not developed with input from end-users, do not support familial communication, and for the most part are not targeted to specific cultural groups [27, 30, 34, 35]. To address this need, we developed a culturally sensitive narrative intervention to facilitate decisions about genetic counseling among Black women at risk for hereditary breast cancer syndromes. The intervention was delivered in conjunction with population-based cancer risk assessment in a clinical setting with medically underserved Black women. The decision aid was grounded in a robust theoretical model of health behavior (the Integrative Model of Behavioral Prediction, IMBP), and development of the intervention was informed by Black women with a family history of breast cancer who were recommended for genetic counseling, by breast cancer advocates, and by primary healthcare providers [36]. Here we report findings from a pilot study with the narrative intervention to demonstrate changes in intention to access genetic counseling and intervention satisfaction. We tested the effect of the intervention on measures of knowledge, self-efficacy, normative beliefs, and intentions to engage in genetic counseling.

## Methods

### Study design

A prospective, pre-post survey study design was used to evaluate baseline and immediate post-intervention intentions, knowledge, and beliefs regarding genetic counseling, as well as to assess satisfaction with the video intervention. The study was registered at Clinicaltrials.gov (NCT04082117) and was approved by the Institutional Review Board at the University of Illinois at Chicago (#2016–1219). All study participants provided written informed consent.

### Study population

The study was conducted in a mammography center at a safety net academic medical center in Chicago, IL, between August 2019 and October 2019. Eligibility criteria included: 1) Black women between the ages of 25 to 69 years old presenting for a screening or diagnostic mammogram; 2) no personal history of breast cancer; 3) never attended an appointment with a genetic counselor; 4) underwent breast cancer risk assessment at the time of the mammogram; and 4) were recommended for genetic counseling based on family history of breast and/or ovarian cancer. Formal power calculations are difficult to perform for pilot studies. A sample size of *n* = 30 is commonly recommended for pilot studies [37]. We followed that practice and enrolled 30 participants.

### Breast Cancer risk assessment

The participating mammography center conducted family history screening for hereditary cancer risk as a routine component of the evaluation for all women undergoing screening or diagnostic breast imaging beginning in 2018. Risk assessment was conducted at the time women checked-in for the mammogram using a software application (CancerIQ®) on tablet computers and patient-reported information on family cancer history. Women who met national guideline criteria for genetic counseling referral [38] due to hereditary breast cancer risk were advised by mammography center staff to obtain a referral from their primary care physician (PCP) to the Hereditary Cancer Clinic at our institution. PCPs were notified of the recommendation for genetic counseling referral. Participants for this study were recruited from the group of women who received a recommendation for genetic counseling as a result of the risk assessment performed at the time of their mammogram.

### Study recruitment and participation

Radiology technicians gave a study recruitment flyer to all women who were recommended for genetic counseling and briefly described the study to eligible women using a recruitment script. Women who expressed interest in participating in the study were directed to a privacy-protected area in the mammography center where research personnel completed eligibility screening and informed consent. Research personnel then administered a pre-viewing survey, played the six-minute narrative decision aid on a laptop computer for the participant to view, and then administered a post-viewing survey. Women completing all study activities received a stipend for research participation.

### Data collection

The pre-viewing survey and post-viewing survey were administered by research personnel using an electronic data capture and management system (REDCap) with an encrypted laptop computer. The initial study protocol included review of the electronic health record 6 months after completion of study recruitment to determine rates of attendance at a genetic counseling session. However, due to clinic restrictions that were initiated in 2020 due to the COVID-19 pandemic, all elective outpatient appointments were cancelled, so the 6-month medical record review was not completed.

### Culturally sensitive decision aid to promote genetic counseling

A culturally sensitive, narrative decision aid was developed by the study team to promote uptake of genetic counseling among Black women at risk for a hereditary breast cancer syndrome [36]. Development of the intervention was guided by the IMBP theoretical framework, which asserts that the primary driver of engaging in a health behavior is forming an intention to do so. The process of forming an intention is determined by three cognitive domains (expected outcomes of engaging in the behavior, cultural norms, and self-efficacy beliefs). The IMBP further proposes that the relationship between intention to engage in a health behavior and carrying through on the intention may be impacted by contextual factors [39]. In order to ensure that the intervention is culturally sensitive for Black women, we employed Kreuter’s framework for achieving cultural appropriateness in health promotion programs [40]. Details about intervention design methodology were previously published [36]. Briefly, phase 1 consisted of in-depth, semi-structured interviews and story circles with Black women recommended for genetic counseling by their PCP based on their family history of breast/ovarian cancer [28, 36]. Importantly, the majority of study participants did not attend a genetic counseling session even though it was recommended by their PCP. One-on-one interviews garnered information about motivating and facilitating factors and barriers to genetic counseling attendance. Story circles sought to understand women’s personal and familial experiences related to breast cancer. A script was developed for the narrative intervention based on themes that emerged from in-depth interviews and story circles. Phase 2 consisted of a series of focus groups conducted with three cohorts: an independent group of Black women with a family history of breast cancer who had not undergone genetic counseling and did not participate in one-on-one interviews, primary care providers at a federally qualified health center, and representatives from breast cancer advocacy organizations. Focus groups provided feedback on the script and the final video production. The decision aid that was produced is a six-minute narrative video that tells the story of an Black women recommended for genetic counseling due to the presence of breast cancer in her family. A link to the narrative intervention is provided at the end of this manuscript.

### Survey instrument

Original and adapted pre- and post- survey questions, guided by the IMBP theoretical constructs, assessed participants’ intentions, knowledge, perceived normative beliefs, barriers, self-efficacy beliefs, attitudes, and environmental constraints. Intentions regarding genetic counseling attendance were measured with six items. Responses for four of these items were measured on a 5-point likert scale ranging from 1 (extremely likely) to 5 (extremely unlikely); one item was open-ended; and one item was single response. Knowledge about genetic counseling was measured by six true/false adapted items [41]. Perceived normative beliefs were measured with two adapted yes/no items [42]. Barriers to genetic counseling were captured by four original items (yes/no). Self-efficacy beliefs were also measured with four adapted items with responses captured by a 5-point likert scale ranging from 1 (strongly agree) to 5 (strongly disagree) [43, 44]. Attitudes about the importance of genetic counseling were measured with one original item using a 10-point likert scale ranging from 0 (not at all important) to 10 (very important). Environmental constraints were only measured at pre-test since the intervention was not designed to impact these factors and were measured with original six true/false items. Nine original questions assessed satisfaction with the video, with responses captures as a 5-point Likert scale ranging from 1 (either strongly agree or extremely likely to 5 (strongly disagree or extremely or unlikely) [45] or as yes/no response for three questions, and one open-ended response.

### Statistical analysis

Demographic characteristics of participants were summarized with descriptive statistics. Survey responses were summarized as frequencies and percentages. Responses of “no” and “not sure” were combined into the same response group. Pre- and post-intervention responses were compared using McNemar’s test for paired data. For data from pre- and post-intervention survey items with a dichotomous response (extremely vs. somewhat likely) *p*-values were computed using McNemar’s test of marginal homogeneity (symmetric table cell proportions). For data from pre- and post-intervention survey items with a 3 × 3 response (extremely likely, somewhat likely, or neither likely nor unlikely) p-values were computed using the generalization of the McNemar’s test, commonly referred to as generalized McNemar’s test or Stuart-Maxwell test for homogeneity of the marginal distributions [46]. Analyses were conducted using SAS version 9.4 (Cary, NC). A two-sided *p*-value less than 0.05 was considered statistically significant.

URL link to the intervention: https://youtu.be/_bR81v_nCaM

## Results

Thirty Black women enrolled in this study and completed all study activities. The median age among participants was 53 years (interquartile range 48–60); ages ranged from 30 to 69.

### Intentions regarding genetic counseling

Before viewing the intervention, 50% of participants indicated that they were extremely likely to make a genetic counseling appointment, compared with 70% of participants after the viewing (*p* = 0.05) (Table 1). After watching the intervention, 50% of study participants indicated that the video changed their mind regarding genetic counseling (Table 2). Additionally, after viewing the video more than half of participants (63.3%) indicated they would make an appointment to speak with a genetic counselor within the next 6 months.

**Table 1:** Pre- and Post-intervention Responses to Items Measuring Intentions Regarding Genetic Counseling (*n* = 30)

Intention Questions, n (%)	Pre-intervention	Post-intervention			***p***-value
		Extremely likely	Somewhat likely	Neither likely nor unlikely	
How likely are you to discuss genetic counseling with your family?	**Extremely likely**	23 (76)	2 (7)	0	1.0^a^
	**Somewhat likely**	2 (7)	3 (10)	0	
	**Neither likely nor unlikely**	0	0	0	
How likely are you to speak with your doctor about genetic counseling?	**Extremely likely**	16 (53)	2 (7)	0	0.10^a^
	**Somewhat likely**	7 (23)	5 (17)	0	
	**Neither likely nor unlikely**	0	0	0	
How likely are you to share information about genetic counseling with others, aside from your family?	**Extremely likely**	17 (57)	1 (3)	0	0.37^b^
	**Somewhat likely**	3 (10)	6 (20)	0	
	**Neither likely nor unlikely**	0	1 (3)	2 (7)	
How likely are you to make an appointment with a genetic counselor?	**Extremely likely**	15 (50)	0	0	**0.05**^b^
	**Somewhat likely**	5 (17)	9 (30)	0	
	**Neither likely nor unlikely**	1 (3)	0	0	

^a^For data from pre- and post-intervention survey items with a dichotomous response (extremely vs. somewhat likely) p-values were computed using McNemar’s test of marginal homogeneity (symmetric table cell proportions)

^b^For data from pre- and post-intervention survey items with a 3 × 3 response (extremely likely, somewhat likely, or neither likely nor unlikely) *p*-values were computed using the generalization of the McNemar’s test, commonly referred to as generalized McNemar’s test or Stuart-Maxwell test for homogeneity of the marginal distributions

**Table 2:** Post-Intervention Satisfaction with the Video Intervention (*n* = 30)

Items	Responses	Overall n (%)
The video was enjoyable to watch.	Strongly agree or agree	29 (96.7)
	Neither agree or disagree	1 (3.3)
	Disagree or Strongly disagree	0
My attention was held throughout the entire video.	Strongly agree or agree	29 (96.7)
	Neither agree or disagree	1 (3.3)
	Disagree or Strongly disagree	0
I felt I could relate to what the actors were saying.	Strongly agree or agree	27 (90.0)
	Neither agree or disagree	2 (6.7)
	Disagree or Strongly disagree	1 (3.3)
I enjoyed the mix of real actors and animations.	Strongly agree or agree	28 (93.3)
	Neither agree or disagree	1 (3.3)
	Disagree or Strongly disagree	0
	Did not respond	1 (3.3)^a^
How likely are you to share this video with your family or friends?	Extremely or somewhat likely	29 (96.7)
	Neither likely or unlikely	0
	Somewhat or extremely unlikely	1 (3.3)
Do you think this video would motivate your loved ones to speak with their doctors about genetic counseling?	Yes	27 (90.0)
	No	0
	Not sure	3 (10.0)
Did this video change your mind about genetic counseling?	Yes	15 (50.0)
	No	15 (50.0)
	Not sure	0
Is there anything you would change about the video?	Yes	4 (13.3)
	No	26 (86.7)
	Not sure	0

^a^percentages do not add up to 100% due to rounding

### Normative beliefs and barriers

At baseline, 77% of participants believed their family would be interested in learning more about genetic counseling because of cancer in their family (Table 3). This increased to 94% (*p* = 0.02) following the intervention. Eleven women (37%) indicated that they anticipated experiencing barriers or would have difficulties attending a genetic counseling session before the intervention. There was a decrease in this proportion following the intervention (20%, *p* = 0.06). Only 7 women responded to the item asking if they believed they could overcome any difficulty they may have in attending a genetic counseling session. At baseline, 3 women responded yes, and 5 women responded yes following the intervention.

**Table 3:** Pre- and Post-intervention Responses to Items Measuring Normative Beliefs, Barriers, and Self-efficacy (*n* = 30)

**Normative Beliefs, n (%)**	**Pre-intervention**	**Post-intervention**		***p*-value**
		**Yes**	**No/Not sure**	
Do you think your family would be interested in learning more about genetic counseling because of cancer in your family?	Yes No/Not sure	23 (77) 5 (17)	0 2 (7)*	**0.02**
Do you believe your family would get genetic counseling if their doctor recommended it to them?	Yes No/Not sure	27 (90) 1 (3)	0 2 (7)	0.32
**Barriers, n (%)**				
Difficulties to make an appointment with a genetic counselor.	Yes No/Not sure	4 (13) 2 (7)	5 (17) 19 (63)	0.26
Difficulties to attend an appointment with a genetic counselor.	Yes No/Not sure	5 (17) 1 (3)	6 (20) 18 (60)	0.06
Difficulties to speak with a doctor about genetic counseling.	Yes No/Not sure	2 (7) 1 (3)	1 (3) 26 (87)	1.0
		**Post-intervention**		***p*-value**
**Self-efficacy** (*How much you agree or disagree with the following statements regarding genetic counseling?)***, n (%)**	**Pre-intervention**	**Strongly agree/agree**	**Neutral or disagree/strongly disagree**	
I am confident that I can pay for genetic counseling services.	Strongly agree/agree Neutral or disagree/strongly disagree	4 (13) 8 (27)	1 (3) 17 (57)	**0.02**
I am confident that I can act on information learned as a result from genetic counseling.	Strongly agree/agree Neutral or disagree/strongly disagree	30 (100) 0	0 0	–
I know who to call to make an appointment for genetic counseling.	Strongly agree/agree Neutral or disagree/strongly disagree	9 (30) 11 (37)	0 10 (33)	**0.001**
I feel comfortable talking with my doctor about genetic counseling.	Strongly agree/agree Neutral or disagree/strongly disagree	29 (97) 1 (3)	0 0	–

*P*-values were computed using McNemar’s test of marginal homogeneity

*percentages do not add up to 100% due to rounding

### Self-efficacy

Before the intervention only 17% of participants were confident that they could pay for genetic counseling services (Table 3). This increased significantly to 40% (*p* = 0.02) following the intervention. The proportion of women indicating that they were confident that they knew who to call to make an appointment to attend a genetic counseling session increased from nine (30%) to twenty women (67%, *p* = 0.001) following the intervention.

### Environmental constraints

When asked about health care in general, 50% of women indicated they often worry about the cost of medical care and 63% (*n* = 19) indicated they did not know how much their medical expenses will cost when seeing a doctor. Despite this, 83% (*n* = 25) indicated they could easily get needed medical care when needed, and over 90% felt comfortable asking their doctor questions and making and getting to and from medical appointments.

### Video satisfaction

The majority of participants enjoyed watching the video and felt they could relate to the actors (Table 2). All but one participant indicated they would share the video with family and friends, and 90% (*n* = 27) of participants thought the video would motivate their loved ones to engage with their doctors about genetic counseling. Four participants indicated they would make changes to the video. Women who did specify suggested changes recommended giving more details about the personal benefit a woman gains from genetic counseling in addition to limitations of genetic counseling.

### Knowledge

The intervention was designed to motivate attendance at a genetic counseling session. As expected, knowledge about genetic counseling did not change significantly after viewing the intervention (Table 4).

**Table 4:** Pre- and Post-intervention Responses to Items Measuring Knowledge of Genetic Counseling

Knowledge Question (correct answer)	Correctly Answered Pre-intervention n (%)	Correctly Answered Post-intervention n (%)	*p*-value
A purpose of genetic counseling is to help people understand their ancestry. **(false)**	4 (13.3)	7 (23.3)	0.08
A purpose of genetic counseling is to provide information about how genetics contributes to health problems. **(true)**	30 (100.0)	30 (100.0)	–
A purpose of genetic counseling is to provide an explanation of treatments for breast cancer. **(false)**	6 (20.0)	5 (16.7)	0.74
A purpose of genetic counseling is to provide information about the chances of you or your family developing breast cancer in the future. **(true)**	30 (100.0)	29 (96.7)	–
A purpose of genetic counseling is to help people understand their options for genetic testing. **(true)**	27 (90.0)	30 (100.0)	–
A purpose of genetic counseling is to provide information and support to individuals with breast cancer in their family. **(true)**	27 (90.0)	28 (93.3)	0.32

*P*-values were computed using McNemar’s test of marginal homogeneity

## Discussion

This study demonstrated changes in intention to attend genetic counseling and satisfaction with a decision aid intervention designed to motivate Black women with hereditary breast cancer risk to attend a genetic counseling appointment. The intervention has now been thoroughly evaluated for cultural appropriateness using qualitative research with focus groups in earlier work [36], and now with a quantitative analysis. Moreover, the intervention significantly increased participants’ intentions to attend a genetic counseling session, which was the primary objective of the intervention. The results of this pilot study underscore the value of multiple qualitative methods (i.e., one-on-one interviews, story circles, and focus groups) and a narrative approach when designing interventions to promote uptake of genomically-informed cancer care in underrepresented racial/ethnic minority groups.

Evidence-based decision aid interventions increase patient knowledge, supplement clinical consultations, improve congruence with patient values, reduce decisional conflicts, and help patients make informed decisions [29, 30, 47, 48]. A recent systematic review that aimed to identify published resources to support decision-making related to *BRCA1* and *BRCA2* genetic testing in women with breast cancer found that all studies examined (*n* = 9) described gains in knowledge about hereditary breast cancer, but lacked a theoretical justification for why or how interventions impacted outcomes [30]. Use of a theoretical framework in decision support tool development can inform which theoretical concepts may be most impactful to target in intervention development and inform how and why specific intervention components may or may not impact outcomes [30]. Guided by the IMBP theoretical framework of health behavior, our study showed that intention may be a specific and key construct to target in interventions designed to support decision-making about genetic services. Additionally, the majority of studies that examined the effectiveness of decision support interventions for women with family histories of breast or ovarian cancer were conducted with primarily non-Hispanic white study participants [30, 33, 48] and few studies have assessed factors that influence decision-making regarding genetic counseling utilization among high-risk Black women. The results presented here confirm the relevance of the cognitive domains in the IMBP for racial/ethnic minority women, and the effectiveness of health behavior interventions that are designed to address beliefs mapping to constructs of that theoretical model. Our study also expands understanding of psychosocial factors that may be salient to this group of focus.

This pilot study has limitations. It was intended to inform the design of a subsequent large scale implementation study, and therefore the sample size was relatively small by design. The sample size resulted in sparse data or zero cells for some survey responses, which precluded us from presenting *p*-values for every pre/post comparison. The intervention was designed for urban Black women with a family history of breast cancer. It is unclear whether this intervention would have the same effect with women from other racial/ethnic groups or in other geographic settings, or with different family cancer histories. Additionally, we were unable to complete planned analysis of genetic counseling uptake at 6 months post-intervention since clinic restrictions resulting from the COVID-19 pandemic prevented women from scheduling a non-urgent appointment. However, it is likely that a multilevel approach that also addresses barriers to scheduling and attending the consultation will be necessary in order to maximize uptake of cancer genetic services among underserved, high-risk Black women. We are testing this hypothesis in a randomized trial (NCT 04378751) that will couple additional evidence-based strategies (patient navigation and transportation assistance) with the decision aid in order to increase utilization of genetic counseling in this patient population. We will also assess implementation feasibility with clinical providers. An additional limitation of this study is that we did not test the effect of the decision aid on diffusion of knowledge about genetic risk and counseling among the social networks of Black women recommended for genetic counseling. This is an important aspect of a population health approach to cancer genetics and risk assessment, since the benefits of population screening for genetic risk can be amplified by identifying and testing at-risk family members of patients found to carry a pathogenic variant (cascade testing). We are addressing this issue in the ongoing randomized trial.

## Conclusion

We developed a culturally tailored narrative decision aid that meets the need for cost-effective interventions to facilitate informed decisions on genetic counseling and testing and communication about heritable cancer risks among medically underserved, Black women. Satisfaction with the intervention among the target audience was very high. The intervention increased self-efficacy and intentions to attend genetic counseling among high risk Black women, and it may facilitate cascade testing among at-risk family members. This intervention warrants further investigation as a component of population-based strategies to identify racial/ethnic minority women with hereditary breast cancer risk in an effort to curtail health disparities that may be perpetuated with advances in precision medicine [27, 36, 49].

## Supplementary Information

**Additional file 1.** Pilot Study Pre and Post Survey.

## Data Availability

The datasets used and/or analyzed during the current study are available from the corresponding author on reasonable request.

## Abbreviations

CGRA: Cancer Genetic Risk Assessment
IMBP: Integrative Model of Behavioral Prediction
NHW: Non-Hispanic White
PCP: Primary Care Provider
USPSTF: U.S. Preventive Services Task Force
