# Supplementary material for: Pilot study of a culturally sensitive intervention to promote genetic counseling for breast cancer risk
Source: BMC Health Serv Res. 2022 Jun 25;22:826. doi: 10.1186/s12913-022-08193-x (PMC9233847; doi:10.1186/s12913-022-08193-x)
Supplement: Supplementary file 1 — Additional file 1. Pilot Study Pre and Post Survey. [file 12913_2022_8193_MOESM1_ESM.pdf]

Pilot Study Survey

Thank you for agreeing to participate in our study. You will be asked a few questions followed by a 6 minutes, then a few more questions. It will take approximately 15 minutes to answer questions and view the video.

Interviewer: Verify that patient is African American?	◯ Yes<br>◯ No
Age:	____________________
Date of Birth:	____________________
Interviewer:<br><br>Is women between the ages of 25-69?	◯ Yes<br>◯ No
Have you ever been diagnosed with breast cancer?	◯ Yes<br>◯ No
Have you ever attended an appointment with a genetic counselor?	◯ Yes<br>◯ No<br>◯ Not sure
Interviewer: Verify that patient is not a prisoner.<br><br>Is patient a prisoner?	◯ Yes<br>◯ No
Interviewer:<br>Did you confirm with radiology technician that patient completed Cancer IQ and patient was determined to be at high-risk for breast cancer?	◯ Yes<br>◯ No

PATIENT IS NOT ELIGIBLE TO PARTICIPATE IN STUDY

Next I will ask you some questions about your thoughts regarding genetic counseling. If you aren't sure what genetic counseling is, just take your best guess. Please answer true or false to the following statements:

	True	False
A purpose of genetic counseling is to help people understand their ancestry.	◯	◯
A purpose of genetic counseling is to provide information about how genetics contributes to health problems.	◯	◯
A purpose of genetic counseling is to provide an explanation of treatments for breast cancer.	◯	◯

A purpose of genetic counseling is to provide information about the chances of you or your family developing breast cancer in the future.	◯	◯
A purpose of genetic counseling is to help people understand their options for genetic testing.	◯	◯
A purpose of genetic counseling is to provide information and support to individuals with breast cancer in their family.	◯	◯

So now, I'll read you a definition of genetic counseling:

"Genetic counseling is when you speak with a counselor about your family's health history to determine if you might benefit from getting your genes tested and learn about different things you can do to help prevent breast cancer."

Based on this:

	0 Not at all important	1	2	3	4	5	6	7	8	9	10 Very important
On a scale from 0 to 10, 0 being Not at all Important and 10 being Very Important, how would you rate the importance of African American women with a family history of breast cancer getting genetic counseling?	◯	◯	◯	◯	◯	◯	◯	◯	◯	◯	◯

For the next few questions, please tell me how likely you are to do the following?

[Show answer choice options card]

How likely are you to discuss genetic counseling with your family?

◯ Extremely likely
◯ Somewhat likely
◯ Neither likely nor unlikely
◯ Somewhat unlikely
◯ Extremely unlikely

How likely are you to speak with your doctor about genetic counseling?

◯ Extremely likely
◯ Somewhat likely
◯ Neither likely nor unlikely
◯ Somewhat unlikely
◯ Extremely unlikely

How likely are you to share information about genetic counseling with others, aside from your family?

◯ Extremely likely
◯ Somewhat likely
◯ Neither likely nor unlikely
◯ Somewhat unlikely
◯ Extremely unlikely

How likely are you to make an appointment with a genetic counselor?

◯ Extremely likely
◯ Somewhat likely
◯ Neither likely nor unlikely
◯ Somewhat unlikely
◯ Extremely unlikely

What makes you feel this way?

____

Please choose the option that is true for you:

"I plan to schedule an appointment to speak with a genetic counselor within the next..."

[Show answer choices on card]

◯ 7 days
◯ 30 days
◯ 2 to 6 months
◯ 6 to 12 months
◯ I'm not sure.
◯ I don't plan to get genetic counseling.

Now I would like you to answer yes or no.

Do you think your family would be interested in learning more about genetic counseling because of cancer in your family?

◯ Yes
◯ No
◯ Not sure

Do you believe your family would get genetic counseling if their doctor recommended it to them?

◯ Yes
◯ No
◯ Not sure

Do you have any challenges or difficulties that would make it hard for you to:

	Yes	No	Not sure
Make an appointment with a genetic counselor	◯	◯	◯
Attend a genetic counseling session	◯	◯	◯
Speak with a doctor about genetic counseling	◯	◯	◯

Do you believe you are able to overcome any difficulty you may have in attending a genetic counseling session?

◯ Yes
◯ No
◯ Not sure

Now, I'd like to know how much you agree or disagree with the following statements regarding genetic counseling:

[Show answer options card]

	Strongly agree	Agree	Neither agree or disagree	Disagree	Strongly disagree
I am confident that I can pay for genetic counseling services.	○	○	○	○	○
I am confident that I can act on information learned as a result from genetic counseling.	○	○	○	○	○
I know who to call to make an appointment for genetic counseling.	○	○	○	○	○
I feel comfortable talking with my doctor about genetic counseling.	○	○	○	○	○

Please answer the following questions by stating whether the statement is true or false for you regarding health care in general.

	True	False	Not sure
I often worry about the cost of my medical care.	○	○	○
I feel comfortable asking my doctor questions about any health issues.	○	○	○
I can easily get to and from medical appointments.	○	○	○
I can easily make appointments with doctors when needed.	○	○	○
I know how much my medical expenses will be when I see a doctor.	○	○	○
I can easily get the medical care I need when I need it.	○	○	○

Now, I would like to show you a 6 minute video about genetic counseling.

Link to Video

Now that you've seen the video, I'd like to ask you some questions on your thoughts about the video.

Please tell us how much you agree or disagree with each statement:

[Show answer responses card]

	Strongly agree	Agree	Neither agree or disagree	Disagree	Strongly disagree
The video was enjoyable to watch.	◯	◯	◯	◯	◯
My attention was held throughout the entire video.	◯	◯	◯	◯	◯
I felt I could relate to what the actors were saying.	◯	◯	◯	◯	◯
I enjoyed the mix of real actors and animations.	◯	◯	◯	◯	◯

How likely are you to share this video with your family or friends?

◯ Extremely likely
◯ Somewhat likely
◯ Neither likely nor unlikely
◯ Somewhat unlikely
◯ Extremely unlikely

Is there anything you would change about the video?

◯ Yes
◯ No
◯ Not sure

If yes, please tell me more.

____

Do you think this video would motivate your loved ones to speak with their doctors about genetic counseling?

◯ Yes
◯ No
◯ Not sure

Did this video change your mind about genetic counseling?

◯ Yes
◯ No
◯ Not sure

Thank you for sharing your thoughts about the video. I only have a few more questions, some of these may sound familiar.

	True	False
A purpose of genetic counseling is to help people understand their ancestry.	◯	◯
A purpose of genetic counseling is to provide information about how genetics contributes to health problems.	◯	◯

A purpose of genetic counseling is to provide an explanation of treatments for breast cancer.	○	○
A purpose of genetic counseling is to provide information about the chances of you or your family developing breast cancer in the future.	○	○
A purpose of genetic counseling is to help people understand their options for genetic testing.	○	○
A purpose of genetic counseling is to provide information and support to individuals with breast cancer in their family.	○	○

	0 Not at all important	1	2	3	4	5	6	7	8	9	10 Very important
On a scale from 0 to 10, 0 being Not at all Important and 10 being Very Important, how would you rate the importance of African American women with a family history of breast cancer getting genetic counseling?	○	○	○	○	○	○	○	○	○	○	○

For the next few questions, please tell me how likely you are to do the following?

[Show answer choice options card]

How likely are you to discuss genetic counseling with your family?

- ○ Extremely likely
- ○ Somewhat likely
- ○ Neither likely nor unlikely
- ○ Somewhat unlikely
- ○ Extremely unlikely

How likely are you to speak with your doctor about genetic counseling?

- ○ Extremely likely
- ○ Somewhat likely
- ○ Neither likely nor unlikely
- ○ Somewhat unlikely
- ○ Extremely unlikely

How likely are you to share information about genetic counseling with others, aside from your family?

- ○ Extremely likely
- ○ Somewhat likely
- ○ Neither likely nor unlikely
- ○ Somewhat unlikely
- ○ Extremely unlikely

How likely are you to make an appointment with a genetic counselor?

- ◯ Extremely likely
- ◯ Somewhat likely
- ◯ Neither likely nor unlikely
- ◯ Somewhat unlikely
- ◯ Extremely unlikely

What make you feel this way?

____

Please choose the option that is true for you:

"I plan to schedule an appointment to speak with a genetic counselor within the next..."

- ◯ 7 days
- ◯ 30 days
- ◯ 2 to 6 months
- ◯ 6 to 12 months
- ◯ I'm not sure.
- ◯ I don't plan to get genetic counseling.

Now, please answer yes or no for the following questions.

Do you think your family would be interested in learning more about genetic counseling because of cancer in your family?

- ◯ Yes
- ◯ No
- ◯ Not sure

Do you believe your family would get genetic counseling if their doctor recommended it to them?

- ◯ Yes
- ◯ No
- ◯ Not sure

Do you have any challenges or difficulties that would make it hard for you to:

	Yes	No	Not sure
Make an appointment with a genetic counselor	◯	◯	◯
Attend a genetic counseling session	◯	◯	◯
Speak with a doctor about genetic counseling	◯	◯	◯

Do you believe you are able to overcome any difficulty you may have in attending a genetic counseling session?

- ◯ Yes
- ◯ No
- ◯ Not sure

Now, I'd like to know how much you agree or disagree with the following statements regarding genetic counseling:

[Show answer options card]

	Strongly agree	Agree	Neither agree or disagree	Disagree	Strongly disagree
I am confident that I can pay for genetic counseling services.	◯	◯	◯	◯	◯

I am confident that I can act on information learned as a result of genetic counseling.	◯	◯	◯	◯	◯
I know who to call to make an appointment for genetic counseling.	◯	◯	◯	◯	◯
I feel comfortable talking with my doctor about genetic counseling.	◯	◯	◯	◯	◯

May we contact you in the future if we have any additional questions or for future studies?

◯ Yes
◯ No

Name: ________________________________

MRN: ________________________________

[Get from EMR]

Phone number: ________________________________

Email address: ________________________________
